# *catena*-Poly[[zinc(II)-bis­[μ-1,3-bis­(imidazol-1-ylmeth­yl)benzene-κ^2^ *N* ^3^:*N* ^3′^]] dinitrate methanol solvate]

**DOI:** 10.1107/S1600536809040203

**Published:** 2009-10-10

**Authors:** Liliana Dobrzańska

**Affiliations:** a Department of Chemistry, University of Stellenbosch, Private Bag X1, Matieland, South Africa

## Abstract

In the title coordination compound, {[Zn(C_14_H_14_N_4_)_2_](NO_3_)_2_·CH_3_OH}_*n*_, the cationic complex forms a looped chain containing 24-membered *M*_2_ *L*_2_ rings. The ligand adopts two distinct conformations that are alternated in subsequent loops. The Zn^II^ ion displays a slightly distorted tetra­hedral geometry being coordinated by four N atoms from four 1,3-bis­(imidazol-1-ylmeth­yl)benzene ligands. The nitrate ions and methanol solvent mol­ecules are located between adjacent double-stranded chains and participate in an extensive net of  O—H⋯O and C—H⋯O hydrogen bonds. The resulting three-dimensional assembly is further stabilized by π–π inter­actions between benzene rings [centroid–centroid distances = 3.878 (2) and 3.853 (2) Å].

## Related literature

For earlier studies on metal complexes of ditopic imidazole-based ligands, see: Dobrzańska *et al.* (2006 ▶, 2007 ▶). For similar one-dimensional double-stranded motifs formed with the title ligand, see: Li & Du (2006 ▶), Dobrzańska *et al.* (2008 ▶). For Zn—N distances in related tetra­hedral zinc(II) complexes, see: Hoskins *et al.* (1997 ▶); Chawla *et al.* (2006 ▶).
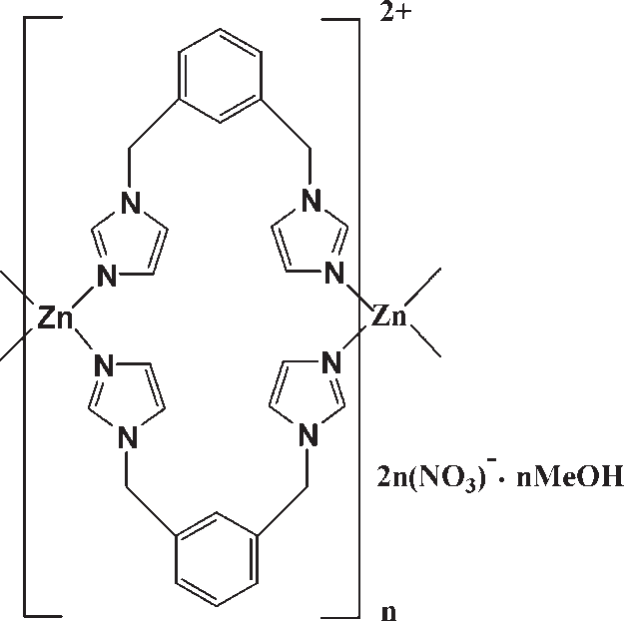


## Experimental

### 

#### Crystal data

[Zn(C_14_H_14_N_4_)_2_](NO_3_)_2_·CH_4_O
*M* *_r_* = 698.02
Triclinic, 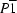

*a* = 9.880 (2) Å
*b* = 12.711 (3) Å
*c* = 13.127 (3) Å
α = 68.798 (4)°
β = 83.167 (4)°
γ = 87.476 (4)°
*V* = 1526.0 (6) Å^3^
*Z* = 2
Mo *K*α radiation
μ = 0.87 mm^−1^
*T* = 100 K
0.21 × 0.10 × 0.10 mm

#### Data collection

Bruker APEX CCD area-detector diffractometer
Absorption correction: multi-scan (*SADABS*; Sheldrick, 1997 ▶) *T*_min_ = 0.838, *T*_max_ = 0.918
17638 measured reflections
7036 independent reflections
4891 reflections with *I* > 2σ(*I*)
*R*_int_ = 0.063

#### Refinement

*R*[*F* ^2^ > 2σ(*F* ^2^)] = 0.056
*wR*(*F* ^2^) = 0.121
*S* = 1.08
7036 reflections
426 parameters
H-atom parameters constrained
Δρ_max_ = 0.65 e Å^−3^
Δρ_min_ = −0.71 e Å^−3^

### 

Data collection: *SMART* (Bruker, 2001 ▶); cell refinement: *SAINT* (Bruker, 2002 ▶); data reduction: *SAINT*; program(s) used to solve structure: *SHELXS97* (Sheldrick, 2008 ▶); program(s) used to refine structure: *SHELXL97* (Sheldrick, 2008 ▶); molecular graphics: *X-SEED* (Barbour 2001 ▶); software used to prepare material for publication: *SHELXL97*.

## Supplementary Material

Crystal structure: contains datablocks I, global. DOI: 10.1107/S1600536809040203/hg2574sup1.cif

Structure factors: contains datablocks I. DOI: 10.1107/S1600536809040203/hg2574Isup2.hkl

Additional supplementary materials:  crystallographic information; 3D view; checkCIF report

## Figures and Tables

**Table:** 

Zn1—N1	1.985 (3)
Zn1—N19	1.986 (3)
Zn1—N35^i^	1.989 (3)
Zn1—N17^ii^	2.009 (3)

**Table:** 

N1—Zn1—N19	109.85 (11)
N1—Zn1—N35^i^	114.88 (11)
N19—Zn1—N35^i^	106.22 (10)
N1—Zn1—N17^ii^	107.39 (10)
N19—Zn1—N17^ii^	110.59 (11)
N35^i^—Zn1—N17^ii^	107.91 (11)

Symmetry codes: (i) 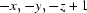; (ii) 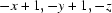.

**Table 2:** Hydrogen-bond geometry (Å, °)

*D*—H⋯*A*	*D*—H	H⋯*A*	*D*⋯*A*	*D*—H⋯*A*
O46—H46⋯O38^iii^	0.84	1.96	2.755 (4)	157
C2—H2⋯O38	0.95	2.40	3.240 (4)	148
C2—H2⋯O40	0.95	2.54	3.420 (4)	154
C4—H4⋯O44	0.95	2.28	3.216 (5)	171
C6—H6*B*⋯O43	0.99	2.33	3.164 (4)	141
C8—H8⋯O46^iv^	0.95	2.54	3.432 (5)	157
C13—H13*B*⋯O38^v^	0.99	2.57	3.350 (5)	136
C20—H20⋯O42^ii^	0.95	2.30	3.085 (4)	139
C20—H20⋯O44^ii^	0.95	2.30	3.207 (5)	160
C22—H22⋯O40^i^	0.95	2.47	3.385 (4)	162
C24—H24*B*⋯O39^i^	0.99	2.51	3.379 (5)	147
C28—H28⋯O42^vi^	0.95	2.59	3.368 (4)	139
C31—H31*A*⋯O46^vi^	0.99	2.42	3.324 (4)	151
C31—H31*B*⋯O43^vi^	0.99	2.49	3.396 (4)	151
C33—H33⋯O43^vi^	0.95	2.57	3.313 (5)	135
C36—H36⋯O40^i^	0.95	2.27	3.135 (4)	151
C10—H10⋯*Cg*1^v^	0.95	2.79	3.539 (4)	136
C15—H15⋯*Cg*2^v^	0.95	2.85	3.762 (4)	162

Symmetry codes: (i) 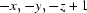; (ii) 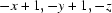; (iii) 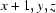; (iv) 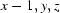; (v) 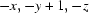; (vi) 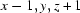. *Cg*1 and *Cg*2 are the centroids of the N1–C5 and C25–C30 rings, respectively.

**Fig. 1.**
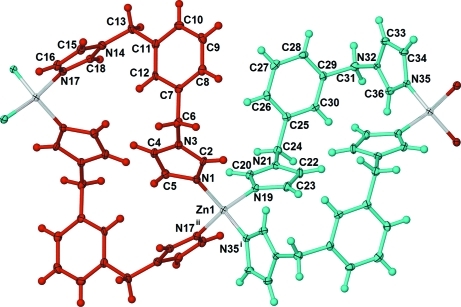
Fragment of an infinite cationic chain, with the atom-numbering scheme. The alternating loops, represented in red and blue, are formed by different conformations of the ligand [symmetry codes:(i) -x, -y, -z + 1; (ii) -x + 1, -y + 1, -z]. Displacement ellipsoids are drawn at the 50% probability level.

**Fig. 2.**
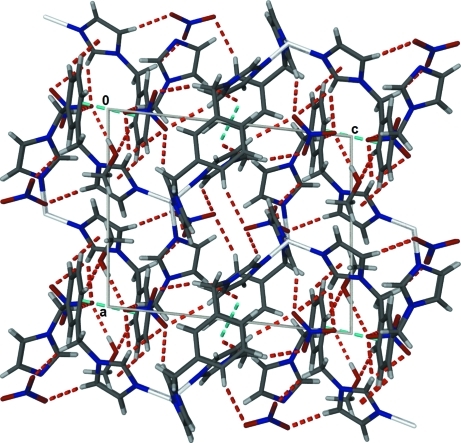
Packing diagram of (I) viewed down [010] showing an extensive net of hydrogen bonds (red dashed lines). Blue dashed lines show π-π stacking interactions.

## supplementary crystallographic information

### Comment

During the course of ongoing studies on metal complexes of ditopic imidazole based ligands (Dobrzańska *et al.*, 2007; Dobrzańska *et al.*, 2006) the title coordination compound (I) was isolated. It consists of infinite cationic double-stranded chains running parallel to [-1 - 1 1], nitrate counterions and methanol molecules. An interesting feature of I is the presence of two different conformers of the 1,3-bis(imidazol-1-ylmethyl)benzene ligand in the asymmetric unit simultaneously with N3—C6—C13—N14 and N21—C24—C31—N32 torsion angles of 70.4 (2) and 12.2 (3)°, respectively. These ligands are bridging Zn^II^ centres by imidazole N-atoms to form cationic looped chains, whereby each loop consists of only one type of conformers (Fig. 1), leading to alternating Zn···Zn distances with values of 9.930 (2) and 9.952 (2) Å. Similar one-dimensional double-stranded motifs were observed in the case of Cu(II) complexes with the same ligand and counterions such as Cl^-^, Br^-^, NO_3_^-^ (see compounds 2, 3, 6, 7 in Dobrzańska *et al.*, 2008) and for a Cd(II) complex with Cl^-^ counterions (Li & Du, 2006). However, in none of these cases two distinct conformers of the ligand were present in a single chain. Furthermore it is worth noting that the corresponding N—C—C—N torsion angles for these reported compounds never exceeded 30°. Each zinc(II) ion in the chain shows a slightly deformed tetrahedral geometry with N—Zn—N bond angles in the range of 106.22 (10) - 114.88 (11)° (Table 1). Zn—N bond lengths are in good agreement with those reported for related tetrahedral zinc(II) complexes (Hoskins *et al.*, 1997; Chawla *et al.*, 2006). The position of the imidazole units in the *M*_2_*L*_2_ rings prevents accommodating any guest molecules inside of the ring spaces formed. Thus nitrate counterions and methanol molecules occupy the spaces between adjacent chains. Both have all of their oxygen atoms participating in an extensive net of hydrogen bonds (Table 2) leading to the formation of a three-dimensional assembly, further stabilized by C—H···π (Table 2) and π-π stacking interactions between benzene rings from adjacent chains [symmetry code: -*x*, 1 - *y*, -*z*, centroid-centroid distance = 3.878 (2) Å with *ca* 1.05 Å slippage for ring C7—C12 and symmetry code: -*x*, 1 - *y*, 1 - *z*, centroid-centroid distance = 3.853 (2) Å with *ca* 2.13 Å slippage for ring C25—C30] (Fig. 2).

### Experimental

A methanolic solution of Zn(NO_3_)_2_^.^4H_2_O was added to a methanolic solution of 1,3-bis(imidazol-1-ylmethyl)benzene in a 1:2 molar ratio. Colorless crystals suitable for single-crystal X-ray diffraction were obtained by slow evaporation.

### Refinement

H atoms were positioned geometrically, with C—H = 0.95 (aromatic), 0.98 (methyl), 0.99 (methylene) and O—H = 0.84 Å, and refined as riding on their parent atoms with *U*_iso_(H) = 1.5*U*_eq_(C) for methyl H and 1.2*U*_eq_(C,*O*) for all other H.

### Crystal data

**Table:**

[Zn(C_14_H_14_N_4_)_2_](NO_3_)_2_·CH_4_O	*Z* = 2
*M**_r_* = 698.02	*F*(000) = 724
Triclinic, *P*1	*D*_x_ = 1.519 Mg m^−^^3^
Hall symbol: -P 1	Mo *K*α radiation, λ = 0.71073 Å
*a* = 9.880 (2) Å	Cell parameters from 2070 reflections
*b* = 12.711 (3) Å	θ = 2.7–22.5°
*c* = 13.127 (3) Å	µ = 0.87 mm^−^^1^
α = 68.798 (4)°	*T* = 100 K
β = 83.167 (4)°	Needle, colorless
γ = 87.476 (4)°	0.21 × 0.10 × 0.10 mm
*V* = 1526.0 (6) Å^3^	

### Data collection

**Table:**

Bruker APEX CCD area-detector diffractometer	7036 independent reflections
Radiation source: fine-focus sealed tube	4891 reflections with *I* > 2σ(*I*)
graphite	*R*_int_ = 0.063
ω scans	θ_max_ = 28.3°, θ_min_ = 1.7°
Absorption correction: multi-scan (*SADABS*; Sheldrick, 1997)	*h* = −12→13
*T*_min_ = 0.838, *T*_max_ = 0.918	*k* = −16→16
17638 measured reflections	*l* = −17→16

### Refinement

**Table:**

Refinement on *F*^2^	Primary atom site location: structure-invariant direct methods
Least-squares matrix: full	Secondary atom site location: difference Fourier map
*R*[*F*^2^ > 2σ(*F*^2^)] = 0.056	Hydrogen site location: inferred from neighbouring sites
*wR*(*F*^2^) = 0.121	H-atom parameters constrained
*S* = 1.08	*w* = 1/[σ^2^(*F*_o_^2^) + (0.0416*P*)^2^] where *P* = (*F*_o_^2^ + 2*F*_c_^2^)/3
7036 reflections	(Δ/σ)_max_ < 0.001
426 parameters	Δρ_max_ = 0.65 e Å^−^^3^
0 restraints	Δρ_min_ = −0.71 e Å^−^^3^

### Special details

**Table:**

Geometry. All e.s.d.'s (except the e.s.d. in the dihedral angle between two l.s. planes) are estimated using the full covariance matrix. The cell e.s.d.'s are taken into account individually in the estimation of e.s.d.'s in distances, angles and torsion angles; correlations between e.s.d.'s in cell parameters are only used when they are defined by crystal symmetry. An approximate (isotropic) treatment of cell e.s.d.'s is used for estimating e.s.d.'s involving l.s. planes.
Refinement. Refinement of *F*^2^ against ALL reflections. The weighted *R*-factor *wR* and goodness of fit *S* are based on *F*^2^, conventional *R*-factors *R* are based on *F*, with *F* set to zero for negative *F*^2^. The threshold expression of *F*^2^ > σ(*F*^2^) is used only for calculating *R*-factors(gt) *etc*. and is not relevant to the choice of reflections for refinement. *R*-factors based on *F*^2^ are statistically about twice as large as those based on *F*, and *R*- factors based on ALL data will be even larger.

### Fractional atomic coordinates and isotropic or equivalent isotropic displacement parameters (Å^2^)

**Table:**

	*x*	*y*	*z*	*U*_iso_*/*U*_eq_	
Zn1	0.43395 (4)	0.12561 (3)	0.25919 (3)	0.01622 (12)	
N1	0.3848 (3)	0.2295 (2)	0.1144 (2)	0.0172 (6)	
C2	0.2852 (3)	0.2180 (3)	0.0597 (3)	0.0180 (7)	
H2	0.2209	0.1582	0.0862	0.022*	
N3	0.2878 (3)	0.3026 (2)	−0.0382 (2)	0.0176 (6)	
C4	0.3955 (3)	0.3723 (3)	−0.0473 (3)	0.0200 (8)	
H4	0.4232	0.4385	−0.1081	0.024*	
C5	0.4534 (3)	0.3278 (3)	0.0471 (3)	0.0203 (8)	
H5	0.5292	0.3587	0.0649	0.024*	
C6	0.1934 (3)	0.3149 (3)	−0.1206 (3)	0.0192 (7)	
H6A	0.1495	0.2411	−0.1038	0.023*	
H6B	0.2467	0.3349	−0.1938	0.023*	
C7	0.0831 (3)	0.4031 (3)	−0.1262 (2)	0.0175 (7)	
C8	−0.0524 (3)	0.3711 (3)	−0.1108 (3)	0.0201 (8)	
H8	−0.0750	0.2939	−0.0912	0.024*	
C9	−0.1554 (3)	0.4517 (3)	−0.1239 (3)	0.0225 (8)	
H9	−0.2480	0.4291	−0.1134	0.027*	
C10	−0.1240 (3)	0.5641 (3)	−0.1520 (3)	0.0206 (8)	
H10	−0.1949	0.6186	−0.1616	0.025*	
C11	0.0113 (3)	0.5976 (3)	−0.1663 (3)	0.0181 (7)	
C12	0.1146 (3)	0.5170 (3)	−0.1536 (3)	0.0167 (7)	
H12	0.2072	0.5395	−0.1635	0.020*	
C13	0.0391 (3)	0.7214 (3)	−0.1923 (3)	0.0219 (8)	
H13A	−0.0297	0.7668	−0.2391	0.026*	
H13B	0.0268	0.7363	−0.1227	0.026*	
N14	0.1753 (3)	0.7599 (2)	−0.2481 (2)	0.0188 (6)	
C15	0.2255 (3)	0.7660 (3)	−0.3530 (3)	0.0205 (8)	
H15	0.1792	0.7456	−0.4026	0.025*	
C16	0.3531 (3)	0.8065 (3)	−0.3707 (3)	0.0204 (8)	
H16	0.4130	0.8197	−0.4363	0.025*	
N17	0.3835 (3)	0.8258 (2)	−0.2793 (2)	0.0169 (6)	
C18	0.2732 (3)	0.7968 (3)	−0.2072 (3)	0.0169 (7)	
H18	0.2649	0.8017	−0.1362	0.020*	
N19	0.2938 (3)	0.1338 (2)	0.3779 (2)	0.0176 (6)	
C20	0.2854 (3)	0.2185 (3)	0.4147 (3)	0.0198 (8)	
H20	0.3369	0.2862	0.3822	0.024*	
N21	0.1943 (3)	0.1956 (2)	0.5041 (2)	0.0167 (6)	
C22	0.1401 (3)	0.0908 (3)	0.5265 (3)	0.0202 (8)	
H22	0.0735	0.0522	0.5852	0.024*	
C23	0.2015 (3)	0.0539 (3)	0.4475 (3)	0.0196 (7)	
H23	0.1837	−0.0161	0.4411	0.024*	
C24	0.1613 (3)	0.2702 (3)	0.5671 (3)	0.0190 (7)	
H24A	0.2286	0.3324	0.5423	0.023*	
H24B	0.1679	0.2270	0.6460	0.023*	
C25	0.0206 (3)	0.3190 (3)	0.5531 (3)	0.0162 (7)	
C26	−0.0099 (3)	0.3968 (3)	0.4524 (3)	0.0178 (7)	
H26	0.0578	0.4180	0.3909	0.021*	
C27	−0.1391 (3)	0.4432 (3)	0.4423 (3)	0.0203 (8)	
H27	−0.1592	0.4966	0.3735	0.024*	
C28	−0.2397 (3)	0.4131 (3)	0.5309 (3)	0.0184 (7)	
H28	−0.3283	0.4452	0.5229	0.022*	
C29	−0.2097 (3)	0.3357 (3)	0.6314 (3)	0.0164 (7)	
C30	−0.0793 (3)	0.2889 (3)	0.6421 (3)	0.0172 (7)	
H30	−0.0589	0.2359	0.7110	0.021*	
C31	−0.3188 (3)	0.2959 (3)	0.7283 (3)	0.0196 (8)	
H31A	−0.2757	0.2690	0.7976	0.024*	
H31B	−0.3799	0.3596	0.7286	0.024*	
N32	−0.3991 (3)	0.2038 (2)	0.7218 (2)	0.0164 (6)	
C33	−0.5207 (3)	0.2139 (3)	0.6789 (3)	0.0208 (8)	
H33	−0.5734	0.2806	0.6523	0.025*	
C34	−0.5507 (3)	0.1099 (3)	0.6821 (3)	0.0209 (8)	
H34	−0.6304	0.0907	0.6587	0.025*	
N35	−0.4477 (3)	0.0362 (2)	0.7246 (2)	0.0161 (6)	
C36	−0.3579 (3)	0.0972 (3)	0.7466 (3)	0.0192 (7)	
H36	−0.2749	0.0686	0.7763	0.023*	
N37	−0.0133 (3)	0.0163 (2)	0.1673 (2)	0.0224 (7)	
O38	0.0067 (3)	0.0871 (2)	0.07062 (19)	0.0298 (6)	
O39	−0.1117 (3)	−0.0482 (2)	0.1936 (2)	0.0395 (7)	
O40	0.0674 (2)	0.01307 (19)	0.23517 (19)	0.0248 (6)	
N41	0.4830 (3)	0.5500 (2)	−0.3360 (2)	0.0205 (6)	
O42	0.5282 (2)	0.6026 (2)	−0.43384 (19)	0.0270 (6)	
O43	0.4038 (2)	0.46743 (19)	−0.31273 (19)	0.0266 (6)	
O44	0.5162 (3)	0.5791 (2)	−0.26093 (19)	0.0345 (7)	
C45	0.6595 (4)	0.0513 (3)	0.0162 (3)	0.0327 (9)	
H45A	0.6559	0.0261	0.0964	0.049*	
H45B	0.6676	−0.0144	−0.0065	0.049*	
H45C	0.5760	0.0929	−0.0074	0.049*	
O46	0.7728 (3)	0.1219 (2)	−0.0325 (2)	0.0484 (8)	
H46	0.8413	0.0930	−0.0003	0.058*	

### Atomic displacement parameters (Å^2^)

**Table:**

	*U*^11^	*U*^22^	*U*^33^	*U*^12^	*U*^13^	*U*^23^
Zn1	0.0157 (2)	0.0134 (2)	0.0171 (2)	−0.00014 (15)	−0.00078 (15)	−0.00285 (16)
N1	0.0146 (15)	0.0137 (15)	0.0216 (15)	−0.0018 (11)	−0.0005 (12)	−0.0046 (12)
C2	0.0216 (18)	0.0119 (17)	0.0161 (17)	−0.0010 (14)	0.0006 (14)	−0.0003 (14)
N3	0.0173 (15)	0.0160 (15)	0.0179 (15)	−0.0024 (12)	−0.0008 (12)	−0.0041 (12)
C4	0.0194 (18)	0.0161 (18)	0.0201 (18)	−0.0039 (14)	−0.0003 (15)	−0.0013 (15)
C5	0.0158 (18)	0.0198 (19)	0.0246 (19)	−0.0047 (14)	−0.0024 (15)	−0.0064 (15)
C6	0.0249 (19)	0.0137 (17)	0.0191 (18)	−0.0034 (14)	−0.0049 (15)	−0.0051 (14)
C7	0.0206 (18)	0.0186 (18)	0.0109 (16)	0.0003 (14)	−0.0019 (14)	−0.0024 (14)
C8	0.0243 (19)	0.0181 (18)	0.0185 (18)	−0.0061 (15)	−0.0018 (15)	−0.0067 (15)
C9	0.0169 (18)	0.028 (2)	0.0231 (19)	−0.0066 (15)	−0.0003 (15)	−0.0096 (16)
C10	0.0206 (19)	0.0217 (19)	0.0189 (18)	0.0037 (15)	−0.0021 (15)	−0.0069 (15)
C11	0.0202 (18)	0.0156 (17)	0.0175 (18)	−0.0029 (14)	0.0015 (14)	−0.0054 (14)
C12	0.0123 (16)	0.0185 (18)	0.0169 (17)	−0.0058 (14)	0.0019 (13)	−0.0040 (14)
C13	0.0152 (18)	0.0197 (19)	0.027 (2)	−0.0040 (14)	0.0032 (15)	−0.0053 (16)
N14	0.0172 (15)	0.0155 (15)	0.0228 (16)	−0.0005 (12)	−0.0008 (12)	−0.0061 (12)
C15	0.026 (2)	0.0167 (18)	0.0176 (18)	−0.0014 (15)	0.0008 (15)	−0.0063 (15)
C16	0.0238 (19)	0.0176 (18)	0.0187 (18)	−0.0044 (15)	−0.0006 (15)	−0.0050 (15)
N17	0.0167 (15)	0.0140 (14)	0.0187 (15)	−0.0013 (12)	−0.0017 (12)	−0.0044 (12)
C18	0.0169 (18)	0.0159 (17)	0.0171 (18)	0.0009 (14)	−0.0015 (14)	−0.0050 (14)
N19	0.0164 (15)	0.0132 (14)	0.0206 (15)	−0.0011 (12)	−0.0018 (12)	−0.0031 (12)
C20	0.0206 (19)	0.0159 (18)	0.0179 (18)	−0.0004 (14)	0.0011 (14)	−0.0011 (15)
N21	0.0141 (14)	0.0157 (15)	0.0197 (15)	0.0008 (11)	−0.0010 (12)	−0.0060 (12)
C22	0.0179 (18)	0.0193 (18)	0.0187 (18)	−0.0077 (14)	0.0012 (14)	−0.0014 (15)
C23	0.0218 (19)	0.0141 (17)	0.0211 (18)	−0.0037 (14)	−0.0029 (15)	−0.0034 (14)
C24	0.0191 (18)	0.0177 (18)	0.0191 (18)	−0.0015 (14)	−0.0023 (14)	−0.0049 (15)
C25	0.0194 (18)	0.0141 (17)	0.0184 (18)	−0.0009 (14)	−0.0020 (14)	−0.0096 (14)
C26	0.0192 (18)	0.0198 (18)	0.0152 (17)	−0.0046 (14)	0.0018 (14)	−0.0080 (14)
C27	0.030 (2)	0.0141 (17)	0.0196 (18)	−0.0021 (15)	−0.0082 (16)	−0.0068 (15)
C28	0.0182 (18)	0.0129 (17)	0.0263 (19)	0.0018 (14)	−0.0081 (15)	−0.0079 (15)
C29	0.0188 (18)	0.0114 (16)	0.0205 (18)	−0.0047 (14)	−0.0007 (14)	−0.0074 (14)
C30	0.0204 (18)	0.0150 (17)	0.0166 (17)	0.0024 (14)	−0.0046 (14)	−0.0055 (14)
C31	0.0206 (18)	0.0153 (18)	0.0244 (19)	−0.0003 (14)	−0.0029 (15)	−0.0087 (15)
N32	0.0135 (14)	0.0112 (14)	0.0207 (15)	0.0005 (11)	0.0000 (11)	−0.0018 (12)
C33	0.0156 (18)	0.0154 (18)	0.029 (2)	0.0008 (14)	−0.0051 (15)	−0.0037 (15)
C34	0.0136 (17)	0.0181 (18)	0.028 (2)	0.0016 (14)	−0.0071 (15)	−0.0035 (15)
N35	0.0136 (14)	0.0140 (14)	0.0174 (15)	0.0001 (11)	−0.0007 (11)	−0.0022 (12)
C36	0.0176 (18)	0.0168 (18)	0.0218 (18)	0.0023 (14)	−0.0037 (14)	−0.0049 (15)
N37	0.0184 (16)	0.0176 (16)	0.0290 (18)	−0.0002 (13)	0.0004 (13)	−0.0067 (14)
O38	0.0385 (16)	0.0260 (14)	0.0173 (13)	−0.0122 (12)	−0.0016 (11)	0.0024 (11)
O39	0.0284 (15)	0.0386 (17)	0.0384 (17)	−0.0185 (13)	−0.0050 (12)	0.0042 (13)
O40	0.0211 (13)	0.0266 (14)	0.0258 (14)	0.0013 (11)	−0.0074 (11)	−0.0069 (11)
N41	0.0179 (16)	0.0152 (15)	0.0276 (17)	0.0027 (12)	−0.0045 (13)	−0.0065 (13)
O42	0.0277 (14)	0.0302 (15)	0.0194 (13)	−0.0086 (11)	0.0036 (11)	−0.0052 (11)
O43	0.0231 (14)	0.0199 (13)	0.0327 (15)	−0.0074 (11)	−0.0049 (11)	−0.0033 (11)
O44	0.0516 (18)	0.0274 (15)	0.0214 (14)	−0.0173 (13)	−0.0071 (13)	−0.0023 (12)
C45	0.028 (2)	0.043 (3)	0.030 (2)	−0.0095 (19)	0.0003 (17)	−0.0171 (19)
O46	0.0335 (17)	0.0474 (19)	0.0427 (18)	−0.0130 (15)	−0.0140 (14)	0.0139 (14)

### Geometric parameters (Å, °)

**Table:**

Zn1—N1	1.985 (3)	N21—C22	1.375 (4)
Zn1—N19	1.986 (3)	N21—C24	1.471 (4)
Zn1—N35^i^	1.989 (3)	C22—C23	1.357 (5)
Zn1—N17^ii^	2.009 (3)	C22—H22	0.9500
N1—C2	1.327 (4)	C23—H23	0.9500
N1—C5	1.391 (4)	C24—C25	1.501 (4)
C2—N3	1.342 (4)	C24—H24A	0.9900
C2—H2	0.9500	C24—H24B	0.9900
N3—C4	1.381 (4)	C25—C30	1.386 (4)
N3—C6	1.473 (4)	C25—C26	1.393 (4)
C4—C5	1.348 (4)	C26—C27	1.382 (4)
C4—H4	0.9500	C26—H26	0.9500
C5—H5	0.9500	C27—C28	1.388 (4)
C6—C7	1.517 (4)	C27—H27	0.9500
C6—H6A	0.9900	C28—C29	1.387 (4)
C6—H6B	0.9900	C28—H28	0.9500
C7—C8	1.386 (4)	C29—C30	1.396 (4)
C7—C12	1.400 (4)	C29—C31	1.513 (4)
C8—C9	1.391 (5)	C30—H30	0.9500
C8—H8	0.9500	C31—N32	1.476 (4)
C9—C10	1.380 (4)	C31—H31A	0.9900
C9—H9	0.9500	C31—H31B	0.9900
C10—C11	1.392 (4)	N32—C36	1.331 (4)
C10—H10	0.9500	N32—C33	1.368 (4)
C11—C12	1.394 (4)	C33—C34	1.352 (4)
C11—C13	1.516 (4)	C33—H33	0.9500
C12—H12	0.9500	C34—N35	1.378 (4)
C13—N14	1.467 (4)	C34—H34	0.9500
C13—H13A	0.9900	N35—C36	1.325 (4)
C13—H13B	0.9900	N35—Zn1^i^	1.989 (3)
N14—C18	1.338 (4)	C36—H36	0.9500
N14—C15	1.383 (4)	N37—O39	1.234 (3)
C15—C16	1.345 (4)	N37—O40	1.253 (3)
C15—H15	0.9500	N37—O38	1.260 (3)
C16—N17	1.377 (4)	N41—O42	1.251 (3)
C16—H16	0.9500	N41—O44	1.252 (3)
N17—C18	1.325 (4)	N41—O43	1.261 (3)
N17—Zn1^ii^	2.009 (3)	C45—O46	1.404 (4)
C18—H18	0.9500	C45—H45A	0.9800
N19—C20	1.327 (4)	C45—H45B	0.9800
N19—C23	1.382 (4)	C45—H45C	0.9800
C20—N21	1.340 (4)	O46—H46	0.8400
C20—H20	0.9500		
			
N1—Zn1—N19	109.85 (11)	N19—C20—H20	124.6
N1—Zn1—N35^i^	114.88 (11)	N21—C20—H20	124.6
N19—Zn1—N35^i^	106.22 (10)	C20—N21—C22	108.1 (3)
N1—Zn1—N17^ii^	107.39 (10)	C20—N21—C24	125.2 (3)
N19—Zn1—N17^ii^	110.59 (11)	C22—N21—C24	126.7 (3)
N35^i^—Zn1—N17^ii^	107.91 (11)	C23—C22—N21	105.8 (3)
C2—N1—C5	105.9 (3)	C23—C22—H22	127.1
C2—N1—Zn1	127.9 (2)	N21—C22—H22	127.1
C5—N1—Zn1	126.2 (2)	C22—C23—N19	109.6 (3)
N1—C2—N3	110.8 (3)	C22—C23—H23	125.2
N1—C2—H2	124.6	N19—C23—H23	125.2
N3—C2—H2	124.6	N21—C24—C25	111.6 (3)
C2—N3—C4	107.7 (3)	N21—C24—H24A	109.3
C2—N3—C6	125.1 (3)	C25—C24—H24A	109.3
C4—N3—C6	127.2 (3)	N21—C24—H24B	109.3
C5—C4—N3	106.4 (3)	C25—C24—H24B	109.3
C5—C4—H4	126.8	H24A—C24—H24B	108.0
N3—C4—H4	126.8	C30—C25—C26	119.4 (3)
C4—C5—N1	109.3 (3)	C30—C25—C24	119.7 (3)
C4—C5—H5	125.4	C26—C25—C24	120.8 (3)
N1—C5—H5	125.4	C27—C26—C25	119.8 (3)
N3—C6—C7	114.4 (3)	C27—C26—H26	120.1
N3—C6—H6A	108.7	C25—C26—H26	120.1
C7—C6—H6A	108.7	C26—C27—C28	121.1 (3)
N3—C6—H6B	108.7	C26—C27—H27	119.5
C7—C6—H6B	108.7	C28—C27—H27	119.5
H6A—C6—H6B	107.6	C29—C28—C27	119.3 (3)
C8—C7—C12	119.2 (3)	C29—C28—H28	120.3
C8—C7—C6	119.0 (3)	C27—C28—H28	120.3
C12—C7—C6	121.6 (3)	C28—C29—C30	119.8 (3)
C7—C8—C9	120.2 (3)	C28—C29—C31	120.9 (3)
C7—C8—H8	119.9	C30—C29—C31	119.2 (3)
C9—C8—H8	119.9	C25—C30—C29	120.6 (3)
C10—C9—C8	120.5 (3)	C25—C30—H30	119.7
C10—C9—H9	119.7	C29—C30—H30	119.7
C8—C9—H9	119.7	N32—C31—C29	110.5 (3)
C9—C10—C11	120.1 (3)	N32—C31—H31A	109.6
C9—C10—H10	120.0	C29—C31—H31A	109.6
C11—C10—H10	120.0	N32—C31—H31B	109.6
C10—C11—C12	119.5 (3)	C29—C31—H31B	109.6
C10—C11—C13	117.6 (3)	H31A—C31—H31B	108.1
C12—C11—C13	122.9 (3)	C36—N32—C33	107.8 (3)
C11—C12—C7	120.5 (3)	C36—N32—C31	125.1 (3)
C11—C12—H12	119.8	C33—N32—C31	126.7 (3)
C7—C12—H12	119.8	C34—C33—N32	106.2 (3)
N14—C13—C11	114.8 (3)	C34—C33—H33	126.9
N14—C13—H13A	108.6	N32—C33—H33	126.9
C11—C13—H13A	108.6	C33—C34—N35	109.4 (3)
N14—C13—H13B	108.6	C33—C34—H34	125.3
C11—C13—H13B	108.6	N35—C34—H34	125.3
H13A—C13—H13B	107.6	C36—N35—C34	105.5 (3)
C18—N14—C15	107.4 (3)	C36—N35—Zn1^i^	127.8 (2)
C18—N14—C13	126.2 (3)	C34—N35—Zn1^i^	126.6 (2)
C15—N14—C13	126.3 (3)	N35—C36—N32	111.1 (3)
C16—C15—N14	106.2 (3)	N35—C36—H36	124.4
C16—C15—H15	126.9	N32—C36—H36	124.4
N14—C15—H15	126.9	O39—N37—O40	121.2 (3)
C15—C16—N17	109.6 (3)	O39—N37—O38	119.7 (3)
C15—C16—H16	125.2	O40—N37—O38	119.2 (3)
N17—C16—H16	125.2	O42—N41—O44	120.1 (3)
C18—N17—C16	106.0 (3)	O42—N41—O43	120.1 (3)
C18—N17—Zn1^ii^	128.5 (2)	O44—N41—O43	119.8 (3)
C16—N17—Zn1^ii^	125.3 (2)	O46—C45—H45A	109.5
N17—C18—N14	110.8 (3)	O46—C45—H45B	109.5
N17—C18—H18	124.6	H45A—C45—H45B	109.5
N14—C18—H18	124.6	O46—C45—H45C	109.5
C20—N19—C23	105.7 (3)	H45A—C45—H45C	109.5
C20—N19—Zn1	123.2 (2)	H45B—C45—H45C	109.5
C23—N19—Zn1	130.5 (2)	C45—O46—H46	109.5
N19—C20—N21	110.7 (3)		
			
N19—Zn1—N1—C2	−71.8 (3)	N1—Zn1—N19—C20	−76.4 (3)
N35^i^—Zn1—N1—C2	47.8 (3)	N35^i^—Zn1—N19—C20	158.8 (2)
N17^ii^—Zn1—N1—C2	167.8 (3)	N17^ii^—Zn1—N19—C20	41.9 (3)
N19—Zn1—N1—C5	111.3 (3)	N1—Zn1—N19—C23	113.2 (3)
N35^i^—Zn1—N1—C5	−129.0 (3)	N35^i^—Zn1—N19—C23	−11.6 (3)
N17^ii^—Zn1—N1—C5	−9.0 (3)	N17^ii^—Zn1—N19—C23	−128.4 (3)
C5—N1—C2—N3	0.5 (4)	C23—N19—C20—N21	0.7 (4)
Zn1—N1—C2—N3	−176.8 (2)	Zn1—N19—C20—N21	−171.7 (2)
N1—C2—N3—C4	0.2 (4)	N19—C20—N21—C22	−0.2 (4)
N1—C2—N3—C6	178.8 (3)	N19—C20—N21—C24	178.8 (3)
C2—N3—C4—C5	−0.9 (4)	C20—N21—C22—C23	−0.4 (4)
C6—N3—C4—C5	−179.4 (3)	C24—N21—C22—C23	−179.4 (3)
N3—C4—C5—N1	1.2 (4)	N21—C22—C23—N19	0.8 (4)
C2—N1—C5—C4	−1.1 (4)	C20—N19—C23—C22	−1.0 (4)
Zn1—N1—C5—C4	176.3 (2)	Zn1—N19—C23—C22	170.7 (2)
C2—N3—C6—C7	103.5 (4)	C20—N21—C24—C25	110.3 (3)
C4—N3—C6—C7	−78.2 (4)	C22—N21—C24—C25	−70.9 (4)
N3—C6—C7—C8	−122.9 (3)	N21—C24—C25—C30	113.7 (3)
N3—C6—C7—C12	61.3 (4)	N21—C24—C25—C26	−68.3 (4)
C12—C7—C8—C9	0.9 (5)	C30—C25—C26—C27	0.0 (5)
C6—C7—C8—C9	−175.0 (3)	C24—C25—C26—C27	−178.0 (3)
C7—C8—C9—C10	−0.2 (5)	C25—C26—C27—C28	−0.3 (5)
C8—C9—C10—C11	−0.7 (5)	C26—C27—C28—C29	0.4 (5)
C9—C10—C11—C12	0.9 (5)	C27—C28—C29—C30	−0.2 (5)
C9—C10—C11—C13	−177.4 (3)	C27—C28—C29—C31	−176.6 (3)
C10—C11—C12—C7	−0.2 (5)	C26—C25—C30—C29	0.1 (5)
C13—C11—C12—C7	178.0 (3)	C24—C25—C30—C29	178.2 (3)
C8—C7—C12—C11	−0.7 (5)	C28—C29—C30—C25	0.0 (5)
C6—C7—C12—C11	175.1 (3)	C31—C29—C30—C25	176.4 (3)
C10—C11—C13—N14	−156.9 (3)	C28—C29—C31—N32	83.1 (4)
C12—C11—C13—N14	24.9 (5)	C30—C29—C31—N32	−93.3 (3)
C11—C13—N14—C18	−114.6 (4)	C29—C31—N32—C36	76.8 (4)
C11—C13—N14—C15	66.3 (4)	C29—C31—N32—C33	−95.7 (4)
C18—N14—C15—C16	0.2 (4)	C36—N32—C33—C34	1.5 (4)
C13—N14—C15—C16	179.4 (3)	C31—N32—C33—C34	175.1 (3)
N14—C15—C16—N17	0.0 (4)	N32—C33—C34—N35	−1.1 (4)
C15—C16—N17—C18	−0.2 (4)	C33—C34—N35—C36	0.3 (4)
C15—C16—N17—Zn1^ii^	174.8 (2)	C33—C34—N35—Zn1^i^	−176.7 (2)
C16—N17—C18—N14	0.3 (3)	C34—N35—C36—N32	0.7 (4)
Zn1^ii^—N17—C18—N14	−174.5 (2)	Zn1^i^—N35—C36—N32	177.6 (2)
C15—N14—C18—N17	−0.3 (4)	C33—N32—C36—N35	−1.4 (4)
C13—N14—C18—N17	−179.6 (3)	C31—N32—C36—N35	−175.1 (3)

Symmetry codes: (i) −*x*, −*y*, −*z*+1; (ii) −*x*+1, −*y*+1, −*z*.

### Hydrogen-bond geometry (Å, °)

**Table:**

*D*—H···*A*	*D*—H	H···*A*	*D*···*A*	*D*—H···*A*
O46—H46···O38^iii^	0.84	1.96	2.755 (4)	157
C2—H2···O38	0.95	2.40	3.240 (4)	148
C2—H2···O40	0.95	2.54	3.420 (4)	154
C4—H4···O44	0.95	2.28	3.216 (5)	171
C6—H6B···O43	0.99	2.33	3.164 (4)	141
C8—H8···O46^iv^	0.95	2.54	3.432 (5)	157
C13—H13B···O38^v^	0.99	2.57	3.350 (5)	136
C20—H20···O42^ii^	0.95	2.30	3.085 (4)	139
C20—H20···O44^ii^	0.95	2.30	3.207 (5)	160
C22—H22···O40^i^	0.95	2.47	3.385 (4)	162
C24—H24B···O39^i^	0.99	2.51	3.379 (5)	147
C28—H28···O42^vi^	0.95	2.59	3.368 (4)	139
C31—H31A···O46^vi^	0.99	2.42	3.324 (4)	151
C31—H31B···O43^vi^	0.99	2.49	3.396 (4)	151
C33—H33···O43^vi^	0.95	2.57	3.313 (5)	135
C36—H36···O40^i^	0.95	2.27	3.135 (4)	151
C10—H10···Cg1^v^	0.95	2.79	3.539 (4)	136
C15—H15···Cg2^v^	0.95	2.85	3.762 (4)	162

Symmetry codes: (iii) *x*+1, *y*, *z*;  (iv) *x*−1, *y*, *z*; (v) −*x*, −*y*+1, −*z*; (ii) −*x*+1, −*y*+1, −*z*; (i) −*x*, −*y*, −*z*+1; (vi) *x*−1, *y*, *z*+1.
